# Left ventricular functional outcomes in His vs. left bundle branch area pacing: Is lead distance important in LBBAP implantation?

**DOI:** 10.1186/s12872-025-05485-y

**Published:** 2026-01-19

**Authors:** Rahadian Oktavianto Pangestu, Raymond Pranata, William Kamarullah, Giky Karwiky, Nuraini Yasmin Kusumawardhani, Chaerul Achmad, Mohammad Iqbal

**Affiliations:** Department of Cardiology and Vascular Medicine, Faculty of Medicine, Universitas Padjadjaran, Hasan Sadikin General Hospital, Bandung, Indonesia

**Keywords:** Conduction system pacing, His bundle pacing, Left bundle branch area pacing, Left ventricular ejection fraction, Lead-to-tricuspid annulus distance

## Abstract

**Background:**

Right ventricular pacing (RVP) has been associated with adverse left ventricular (LV) remodeling due to electrical and mechanical dyssynchrony. Conduction system pacing (CSP), including His bundle pacing (HBP) and left bundle branch area pacing (LBBAP), has emerged as a more physiological alternative to preserve ventricular function. However, comparative data between these CSP modalities particularly regarding the influence of lead-to-tricuspid annulus distance (LTTAD) in LBBAP remain limited.

**Objective:**

To compare LV functional outcomes between HBP and LBBAP, and to assess the impact of LTTAD on LVEF improvement among LBBAP patients.

**Methods:**

This prospective observational study included 51 patients who underwent CSP (28 with HBP and 23 with LBBAP). Echocardiographic parameters, including LVEF and global longitudinal strain (GLS), were measured at baseline and 12 months post-implantation. Subgroup analysis in the LBBAP group evaluated the relationship between LTTAD and LVEF improvement, using a ≥ 5% increase in LVEF as the threshold.

**Results:**

Both groups demonstrated significant improvement in LVEF over 12 months, with greater improvement in the LBBAP group (+ 7.69% vs. +3.00%, *P* = 0.043). GLS improvement was also more pronounced in the LBBAP group (*P* < 0.001). Subgroup analysis revealed that patients with LTTAD ≥ 21.5 mm experienced improvements in left ventricular (LV) function that were not inferior compared to those with shorter LTTAD.

**Conclusion:**

Both HBP and LBBAP improve LV function, with LBBAP demonstrating more favorable pacing parameters. Distal lead positioning in LBBAP (LTTAD ≥ 21.5 mm) is not associated with deterioration in ventricular synchrony, supporting its broader use in CSP strategies.

## Introduction

Right ventricular pacing (RVP) has long been the standard approach for patients requiring permanent cardiac pacing. However, chronic RVP is associated with non-physiologic ventricular activation, leading to electrical and mechanical dyssynchrony, which can contribute to pacing-induced cardiomyopathy and adverse remodelling of the left ventricle (LV) [1, 2]. To overcome these limitations, conduction system pacing (CSP) techniques such as His bundle pacing (HBP) and left bundle branch area pacing (LBBAP) have emerged as promising physiological pacing strategies. These modalities directly engage the native His–Purkinje system to restore synchronous ventricular activation and preserve LV function [3, 4]. HBP captures the His bundle at its origin and has been shown to maintain left ventricular ejection fraction (LVEF) and mechanical synchrony more effectively than RVP. However, HBP is technically challenging, with limitations including high and unstable pacing thresholds, lead dislodgement, and lower success rates in correcting bundle branch blocks [1, 5]. LBBAP, by contrast, involves pacing the left bundle branch or adjacent septal conduction fibres via transseptal lead implantation. It offers more stable thresholds, higher R-wave sensing, and broader applicability in patients with infranodal conduction disease or heart failure [6, 7]. 

Several refinements to LBBAP implantation technique have been proposed, including the distinction between proximal left bundle branch pacing (LBBP), left fascicular pacing (LFP), and left ventricular septal pacing (LVSP), each of which differs in lead depth, capture pattern, and electrophysiological synchrony. These subtypes reflect the heterogeneity of pacing sites within the left conduction system and raise the possibility that the exact anatomical position of the lead may influence ventricular activation and clinical outcomes [8]. Distal implantation in the left conduction system is associated with delayed activation of the right bundle branch (RBB), which is feared to cause interventricular dyssynchrony [9]. However, the effect of delayed activation induced by LBBAP on this synchrony remains unclear [10]. A concern particularly associated with more distal LBBAP implantation is delayed activation of the right bundle branch, which may theoretically generate interventricular mechanical dyssynchrony. However, data regarding whether distal pacing truly impairs LV mechanics remain inconclusive [11, 12]. 

Conversely, more proximal implantation may place the lead closer to the tricuspid annulus, potentially causing leaflet interference and worsening tricuspid regurgitation (TR). This has introduced interest in the Lead-to-Tricuspid Annulus Distance (LTTAD), defined as the spatial distance between the pacing lead tip and the tricuspid valve annulus, as a potential determinant of both tricuspid valve function and long-term left ventricular performance. Previous reports have linked a shorter LTTAD to TR deterioration, yet whether a more distal position compromises mechanical synchrony has not been adequately evaluated [12]. 

Given these conflicting considerations, a knowledge gap persists regarding whether LTTAD affects functional outcomes in patients undergoing LBBAP. Therefore, the present study aimed not only to compare left ventricular function between HBP and LBBAP, but also to investigate whether LTTAD influences LVEF and GLS improvement over a 12-month follow-up period.

## Methods

### Data collection

Patient data were collected from medical records and local registries and comprised demographic characteristics, comorbidities, NYHA classification, echocardiographic parameters, types of permanent pacemaker (PPM) implanted, and pacing parameters at Dr. Hasan Sadikin General Hospital, Bandung, Indonesia, over a five-year span from 2020 to 2025. The PPM types included HBP or LBBAP, which used either single or dual chamber pacing, the echocardiographic parameters before and twelve months after PPM implantation included LVEF, PSD, LVMD, LAVI, LTTAD and GLS. The standard transthoracic echocardiography study evaluated cardiac dimensions, and Biplane Simpson’s rule was used to assess the LVEF. All echocardiographic examinations were performed using a commercially available ultrasound system (Vivid S70, GE Healthcare, Horten, Norway) equipped with an adult phased‑array transducer for transthoracic imaging. Standard parasternal and apical views were obtained according to the American Society of Echocardiography guidelines. Quantitative measurements (LVEF, GLS, PSD, LVMD, LAVI, and LTTAD) were performed offline using vendor‑specific software (EchoPAC, GE Healthcare). All measurements were averaged over three consecutive cardiac cycles in sinus rhythm and over five cycles in atrial fibrillation. Analyses were performed by two independent experienced echocardiographers who were blinded to pacing modality and clinical outcomes.

### Study design

This study is a prospective, observational, comparative investigation conducted at Dr. Hasan Sadikin General Hospital, Bandung, Indonesia, over a five-year period (2020–2025). The study aimed to evaluate differences in left ventricular (LV) functional outcomes between His bundle pacing (HBP) and left bundle branch area pacing (LBBAP), and to assess, within the LBBAP cohort, the association between lead position—expressed as the lead-to-tricuspid annulus distance (LTTAD)—and changes in LV function over 12 months.

All data were obtained from a prospective institutional registry of conduction system pacing procedures and supplemented by review of electronic medical records. Baseline and follow-up transthoracic echocardiography, as well as device interrogation parameters (pacing threshold, impedance, sensing, and QRS duration), were routinely collected at implantation and at approximately 12 months.

### Patient selection

This study employed a prospective design with a total sampling method, in which all consecutive patients who underwent HBP or LBBAP at our centre during the study period and met the eligibility criteria were considered for inclusion.

Inclusion criteria were:

1. Age ≥ 18 years.
2. Undergoing de novo implantation of a permanent pacemaker using either HBP or LBBAP as the ventricular pacing strategy.
3. Availability of baseline transthoracic echocardiography within 3 months prior to implantation.
4. Availability of follow-up transthoracic echocardiography at approximately 12 months post-implantation.
5. Availability of complete device interrogation data at follow-up, including pacing threshold, impedance, sensing, and paced QRS duration.

Exclusion criteria were:

1. Prior implantation of a permanent pacemaker, implantable cardioverter-defibrillator, or cardiac resynchronization therapy device.
2. Lead dislodgement or revision of the ventricular lead before the 12-month follow-up.
3. Follow-up duration of less than 12 months or incomplete follow-up data.
4. Terminal condition or severe systemic illness that could interfere with the interpretation of cardiac function (e.g., metastatic cancer, severe sepsis).
5. Absence of an adequate evaluation of lead position relative to the tricuspid annulus (LTTAD data not available or non-measurable).

Indications for permanent pacemaker implantation followed contemporary guideline-based recommendations and included symptomatic bradycardia due to sinus node dysfunction, high-grade atrioventricular block, and atrial fibrillation with slow ventricular response. The decision to perform HBP or LBBAP, and to use single- or dual-chamber systems, was made by the implanting electrophysiologist based on the underlying conduction abnormality, anticipated ventricular pacing burden, venous access, and anatomical considerations.

### Pacing procedures and definitions

All pacemaker implantations were performed by experienced electrophysiologists using standard fluoroscopic and electrocardiographic guidance. Conduction system pacing was achieved using either HBP or LBBAP, according to individual clinical indications and operator preference.

### His bundle pacing (HBP)

For HBP, an active‑fixation lead was positioned at the His bundle region on the anteroseptal tricuspid annulus. The target site was identified by mapping a discrete His potential on the intracardiac electrogram and by fluoroscopic positioning in the right anterior oblique and left anterior oblique projections. HBP was considered successful when pacing at low output produced a QRS morphology that closely resembled or was narrower than the intrinsic QRS, with shortening of the His–ventricular interval and either selective or non‑selective His capture, in accordance with current consensus definitions [10, 13]. 

### Left bundle branch area pacing (LBBAP)

For LBBAP, an active‑fixation lead was screwed deep into the right ventricular septum toward the left side, approximately 1–2 cm apical to the His bundle region, guided by fluoroscopy and paced QRS morphology. LBBAP was defined as pacing within the left bundle branch region or adjacent septal conduction system, typically resulting in a right bundle branch block–like pattern in lead V1 with a relatively narrow QRS complex. Capture of the left conduction system was confirmed by a combination of paced QRS morphology, abrupt shortening of left ventricular activation time in the precordial leads, and stability of these findings at different pacing outputs, in line with contemporary recommendations [4, 10, 13]. 

### Lead‑to‑tricuspid annulus distance (LTTAD)

**Fig. 1:**
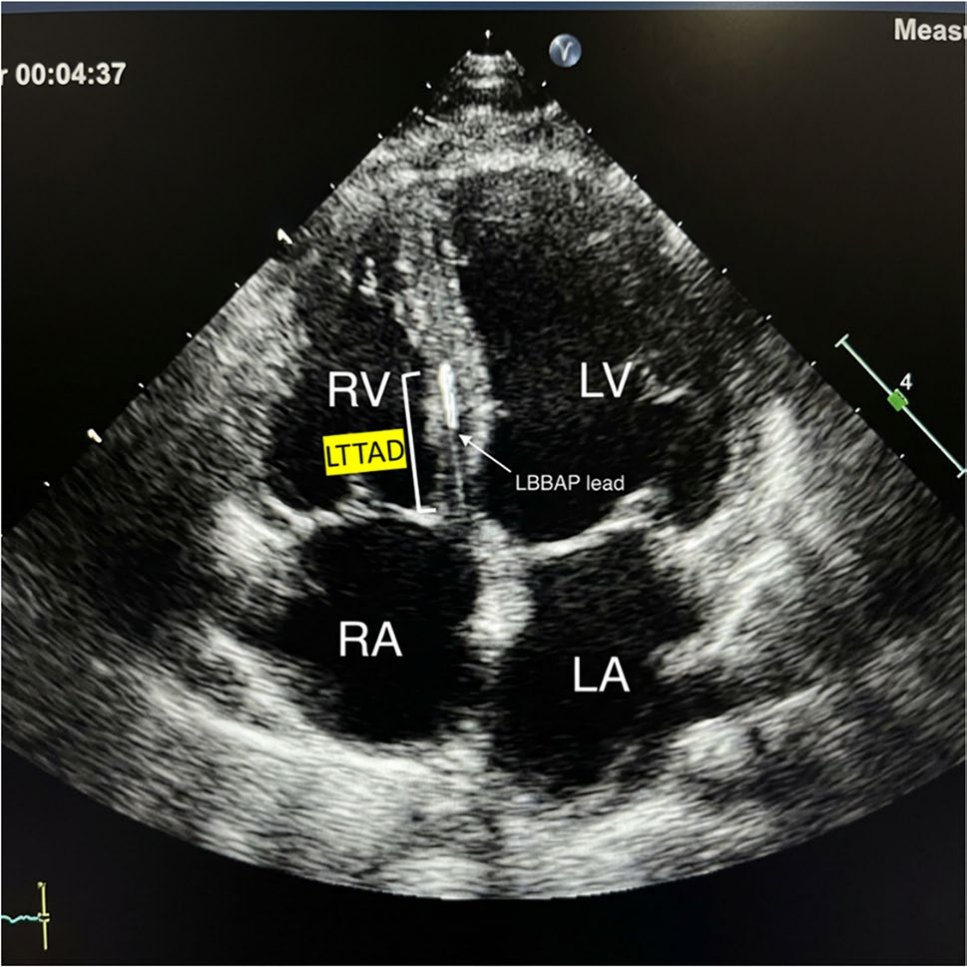
Measurement of echocardiographic distance between lead-implanted site and the tricuspid valve annulus (LTTAD)

LTTAD was defined as the linear distance between the tip of the LBBAP lead and the tricuspid valve annulus along the course of the interventricular septum. In this study, LTTAD was measured using two‑dimensional transthoracic echocardiography in the apical four‑chamber view. The distance from the ventricular portion of the tricuspid annular plane to the pacing lead tip was measured at end‑diastole and averaged over three consecutive cardiac cycles. All measurements were performed offline by experienced echocardiographers who were blinded to clinical outcomes [12]. Fig. 1.

### Statistical analysis

The Shapiro–Wilk test was used to assess the distribution of continuous variables. Categorical variables were expressed as counts and percentages, whereas continuous variables were expressed as mean ± standard deviation for normally distributed data or median (interquartile range) for non‑normally distributed data. Baseline comparisons between HBP and LBBAP groups were performed using the independent samples t‑test for normally distributed continuous variables or the Mann-Whitney U test for non‑normally distributed variables, and the χ² test or Fisher’s exact test for categorical variables, as appropriate. For variables measured at two time points (baseline and 12 months), we performed both within‑group and between‑group comparisons:

Within‑group pre–post comparisons (e.g., LVEF, LV GLS, QRS duration, PSD, LVMD, and LAVI) were assessed using the paired t‑test for normally distributed variables or the Wilcoxon signed‑rank test for non‑normally distributed variables.

To compare the magnitude of change between HBP and LBBAP, we calculated the absolute change (Δ = value at 12 months − baseline value) for each parameter and compared ΔLVEF, ΔGLS, ΔQRS duration, ΔPSD, and ΔLVMD between groups using the independent samples t‑test or Mann–Whitney U test, according to data distribution.

In the LBBAP subgroup analysis, patients were first categorized according to LVEF improvement (≥ 5% vs. < 5%), and clinical, echocardiographic, and device parameters were compared between groups using the same tests described above. Receiver operating characteristic (ROC) curve analysis was then used to determine the optimal cut‑off value of lead‑to‑tricuspid annulus distance (LTTAD) for predicting an LVEF improvement ≥ 5%. The cut‑off was defined using the Youden index (sensitivity + specificity − 1) and was subsequently used to classify patients into two groups (LTTAD < cut‑off vs. LTTAD ≥ cut‑off) for further comparisons.

To identify independent predictors of LVEF improvement ≥ 5%, we performed multivariable logistic regression analysis with LVEF improvement ≥ 5% (yes/no) as the dependent variable. Because of the limited sample size, we constructed a series of parsimonious, nested models with a predefined set of clinically relevant covariates:

Model 1 (core model): included baseline QRS duration and baseline LVEF, as the principal electrophysiological and functional variables of interest.

Model 2 (clinical model): included all variables from Model 1 and was additionally adjusted for baseline clinical covariates: age, hypertension, diabetes mellitus, and coronary artery disease.

Model 3 (clinical + technical model): included all variables from Model 2 and further adjusted for device‑related parameters, specifically ventricular pacing burden (%) and device type (single‑ vs. dual‑chamber system).

Model 4 (full model): included all variables from Model 3 and additionally incorporated pacing modality (LBBAP vs. HBP) as a categorical variable.

For each model, odds ratios (ORs) with 95% confidence intervals (CIs) were reported. All tests were two‑sided, and a p‑value < 0.05 was considered statistically significant. Statistical analyses were performed using SPSS version 26.0 (IBM Corp., Armonk, NY, USA).

## Results

### Baseline characteristics

Baseline characteristics of patients in the HBP (*n* = 28) and LBBAP (*n* = 23) groups are presented in Table 1. In general, both groups had comparable clinical profiles in terms of age, sex, NYHA classification, and comorbidities such as hypertension, diabetes, and coronary artery disease. There were also no significant differences in pacing indications or device types. However, a significant difference was observed in baseline left ventricular function, with the LBBAP group demonstrating a lower LVEF compared to the HBP group (44.39 ± 11.80% vs. 52.78 ± 13.26%, *P* = 0.038). Other echocardiographic parameters such as LV GLS, PSD, LVMD, and LAVI did not differ significantly between the groups. In contrast, technical device parameters revealed notable differences. The LBBAP group had a significantly longer QRS duration at follow-up compared to the HBP group (*P* < 0.001), as well as higher impedance and R-wave sensing values (*P* < 0.001). Pacing thresholds were significantly lower in the LBBAP group at both implantation and follow-up (*P* < 0.001). These findings reflect distinct electrophysiological profiles and implantation characteristics between the two pacing techniques.

**Table 1:** Baseline characteristics

Characteristics		Total *n* = 51		HBP *n* = 28		LBBAP *n* = 23		*p*	
Age (year), mean ± SD		56.3 ± 16.2		57.9 ± 15.8		56.1 ± 15.4		0.732	
Sex. frequency (%)									
Male		28 (54.9)		15 (53.6)		13 (56.5)		0.851	
Female		23 (45.1)		13 (46.4)		10 (43.5)		0.853	
Comorbidities, frequency (%)									
Hypertension		35 (68.6)		20 (71.4)		15 (65.2)		0.601	
Diabetes mellitus		18 (35.3%)		10 (35.7%)		8 (34.8%)		0.914	
Chronic kidney disease		10 (19.6%)		6 (21.4%)		4 (17.4%)		0.723	
Coronary artery disease		12 (23.5%)		7 (25.0%)		5 (21.7%)		0.762	
Heart Failure		26 (51.0%)		14 (50.0%)		12 (52.2%)		0.902	
NYHA Class ≥ II (%)		20 (39.2%)		11 (39.3%)		9 (39.1%)		0.982	
Indiication & device type (%)									
TAVB		33 (64.7%)		18 (64.3%)		15 (65.2%)		0.981	
SSS/SND		10 (19.6%)		6 (21.4%)		4 (17.4%)		0.742	
AF with SVR		5 (9.8%)		3 (10.7%)		2 (9.5%)		0.903	
Other bradycardia indications		3 (5.9%)		0		(7.9%)		0.835	
Single chamber device		20 (39.2%)		8 (57.1%)		12 (60.9%)		0.833	
Dual chamber device		31 (60.8%)		18 (64.3%)		13 (61.9%)		0.712	
Echocardiography, mean ± SD									
LVEF (%)		51.38 ± 12.45		52.78 ± 13.26		44.39 ± 11.80		**0.038**	
LV GLS (-%)		−13.84 ± 4.14		−14.93 ± 4.49		−12.91 ± 4.28		0.107	
PSD (ms)		92.97 ± 49.58		78.89 ± 43.69		106.44 ± 52.03		0.061	
LVMD (ms )		83.49 ± 43.16		73.79 ± 35.19		92.80 ± 48.53		0.123	
LVEDD (mm)		54.75 ± 5.52		56.00 ± 4.92		54.57 ± 5.94		0.829	
LAVI (ml/m2)		36.00 ± 11.59		38.50 ± 4.95		35.29 ± 13.12		0.754	
Implanted pacemaker parameters, mean ± SD									
Baseline QRS duration (ms)		123.01 ± 33.01		120.57 ± 32.35		124.95 ± 33.76		0.561	
Follow-up QRS duration (ms)		118.67 ± 15.15		111.68 ± 16.09		124.23 ± 11.86		**< 0.001**	
VP burden > 20% (%)		40 (78.4%)		21 (75%)		19 (82.6%)		0.858	
R wave at implantation (mV)		9.27 ± 5.99		5.59 ± 4.05		12.20 ± 5.69		**< 0.001**	
Implantation impedance (Ω)		713.71 ± 182.93		583.57 ± 135.18		817.23 ± 146.65		**< 0.001**	
Threshold at implantation (V at 0.4 ms)		0.89 ± 0.51		1.14 ± 0.62		0.69 ± 0.29		**< 0.001**	
Threshold at follow-up (V at 0.4 ms)		0.92 ± 0.79		1.32 ± 1.04		0.61 ± 0.23		**< 0.001**	

*TAVB* Total Atrioventricular Block,* SND* Sinus Node Dysfunction, *SSS* Sick Sinus Syndrome, *LVEF* Left Ventricular Ejection Fraction, *LV GLS* Left Ventricular Global Longitudinal Strain, *AF with SVR* Atrial fibrillation with Slow Ventricular Response, *LAVI* Left Atrium Volume Index, *LVEDD* Left Ventricular End Diastolic Diameter, *PSD* Peak Strain Dispersion, *LVMD *Left Ventricular Mechanical Dyssynchrony

### Dynamic evaluation of LVEF and LV strain after implantation

The development of left ventricular function was evaluated based on LVEF and LV GLS from baseline to 12 months post-implantation (Table 2). Both groups showed a trend toward improved LV function, but the LBBAP group experienced a greater increase. An improvement in LVEF from pre-implantation to 12 months was recorded as + 7.69 ± 9.83% in the LBBAP group, compared to + 3.00 ± 7.54% in the HBP group, with a statistically significant difference (*P* = 0.043). Changes in LV GLS also showed a greater improvement in the LBBAP group (Δ0–12 months: − 3.03 ± 2.17% vs. − 0.86 ± 2.89%; *P* < 0.001). Parameters of ventricular synchrony, such as PSD and LVMD, decreased in both groups, indicating improved mechanical synchrony, although the reduction did not reach statistical significance.

**Table 2:** Evolution of Left Ventricular Functional Parameters Over Time in HBP and LBBAP Groups

Parameters	Time	HBP *n* = 28	LBBAP *n* = 23	Mean difference and standard deviation	*p*
LVEF (%)					
	Before implantation	52.78 ± 13.26	44.39 ± 11.80	8.39 ± 3.24	**0.012**
	3 months	54.39 ± 11.84	51.91 ± 11.53	2.48 ± 3.02	0.414
	12 months	55.79 ± 11.58	52.09 ± 12.12	3.70 ± 3.06	0.232
Δ 0–3 months		+ 1.61 ± 6.43	+ 7.52 ± 8.64	−5.91 ± 1.97	**0.004**
Δ 0–12 months		+ 3.00 ± 7.54	+ 7.69 ± 9.83	−4.69 ± 2.26	**0.043**
Δ 3–12 months		+ 1.39 ± 7.51	+ 0.17 ± 4.47	1.22 ± 1.60	0.448
LV GLS (%)	Before implantation	−14.93 ± 4.49	−12.91 ± 4.28	−2.02 ± 4.39	0.113
	3 months	−14.23 ± 4.77	−14.35 ± 3.68	+ 0.12 ± 4.26	0.076
	12 months	−17.78 ± 3.65	−15.94 ± 3.42	−1.84 ± 3.54	0.053
Δ 0–3 months		0.63 ± 1.29	−1.44 ± 1.42	+ 2.07 ± 1.83	**0.010**
Δ 0–12 months		0.86 ± 2.89	−3.03 ± 2.17	+ 3.89 ± 2.69	**< 0.001**
Δ 3–12 months		0.24 ± 3.29	−1.59 ± 1.28	+ 1.83 ± 2.39	0.078
PSD (ms)					
	Before implantation	78.89 ± 43.69	106.44 ± 52.03	−27.55 ± 47.94	0.051
	12 months	52.98 ± 28.13	72.68 ± 32.11	−19.70 ± 30.36	0.079
Δ 0–12 months		−25.91 ± 25.76	−33.76 ± 26.91	+ 7.85 ± 26.33	0.287
LVMD (ms)	Before implantation	73.79 ± 35.19	92.80 ± 48.53	−19.01 ± 42.40	0.119
	12 months	50.13 ± 24.19	69.04 ± 31.85	−18.91 ± 28.26	0.092
Δ 0–12 months		−23.66 ± 23.01	−23.76 ± 22.42	+ 0.10 ± 22.72	0.986

### Factors associated with LVEF Improvement ≥ 5%

A subgroup analysis of the LBBAP group was conducted to evaluate factors associated with LVEF improvement ≥ 5% (Table 3). No significant differences were found in clinical characteristics such as age, sex, comorbidities, or technical parameters including QRS duration, ventricular pacing (VP) burden, threshold, or impedance. However, a significant difference was observed in the Lead to Tricuspid Annulus Distance (LTTAD), where patients with LVEF improvement ≥ 5% had a longer LTTAD (24.2 ± 2.7 mm vs. 17.5 ± 2.7 mm; *P* = 0.002). This indicates that a more distal lead implantation is not necessarily inferior to a more proximal lead in LBBAP. Distal leads are often associated with poorer electrophysiological synchrony. This finding was supported by the analysis in Table 4, which showed that an LTTAD ≥ 21.5 mm was associated with greater LVEF improvement and LV GLS recovery over 12 months (*P* = 0.009 and *P* < 0.001, respectively). In addition, patients with an LVEF improvement ≥ 5% also showed a numerically greater reduction in PSD and LVMD compared to the group with an LVEF improvement < 5%, although these differences did not reach statistical significance. The P-values for ΔPSD and ΔLVMD were 0.053 and 0.058, respectively, indicating a trend toward a meaningful difference. This suggests that improvement in intraventricular synchrony is positively correlated with the increase in LVEF.

**Table 3:** Factors Associated with LVEF Improvement ≥5% in patients undergoing LBBAP

Parameters	LVEF Improvement ≥ 5% (*n* = 13)	LVEF Improvement < 5% (*n* = 10)	*p*
Clinical characteristics			
Age (year)	56.3 ± 16.2	52.7 ± 14.8	0.212
Sex (%)			
Male	7 (53.8%)	6 (60.0%)	0.764
Female	6 (46.2%)	4 (40.0%)	0.910
Comorbids, frequency (%)			
Hypertension	9 (69.2%)	7 (70.0%)	0.961
Diabetes mellitus	4 (30.8%)	4 (40.0%)	0.645
Chronic kidney disease	3 (23.1%)	3 (30.0%)	0.705
Coronary artery disease	3 (23.1%)	4 (40.0%)	0.404
Heart Failure	6 (46.2%)	6 (60.0%)	0.499
NYHA Class ≥ II (%)	5 (38.5%)	6 (60.0%)	0.287
Indiication & device type (%)			
TAVB	8 (61.5%)	6 (60.0%)	0.945
SSS/SND	3 (23.1%)	2 (20.0%)	0.545
AF with SVR	1 (7.7%)	1 (10.0%)	0.980
Other bradycardia indications	1 (7.7%)	1 (10.0%)	0.980
Single chamber devie	7 (53.8%)	4 (40.0%)	0.509
Dual chamber device	8 (61.5%)	6 (60.0%)	0.945
Implanted pacemaker parameters			
Baseline QRS duration (ms)	123.01 ± 33.01	124.33 ± 29.15	0.420
Follow-up QRS duration (ms)	123.67 ± 15.15	124.11 ± 18.42	0.208
VP burden > 20% (%)	11 (84.6%)	7 (70.0%)	0.391
R wave at implantation (mV)	9.27 ± 5.99	8.45 ± 4.78	0.612
Implantation impedance (Ω)	713.71 ± 182.93	692.33 ± 174.85	0.746
Threshold at implantation (V at 0.4 ms)	0.89 ± 0.51	0.91 ± 0.42	0.912
Threshold at follow-up (V at 0.4 ms)	0.92 ± 0.79	0.95 ± 0.63	0.874
LTTAD (mm)	24.2 ± 2.7	17.5 ± 2.7	**0.002**
Echocardiography parameters			
ΔPSD (ms)	−39.2 ± 25.0	−28.1 ± 27.3	0.053
ΔLVMD (ms)	−27.9 ± 22.6	−19.4 ± 21.9	0.058

**Table 4:** Comparison of Left Ventricular Function Based on LTTAD in LBBAP Patients

Parameters	LTTAD < 21.5 mm (mean ± SD)	LTTAD ≥ 21.5 mm (mean ± SD)	*p*
ΔLVEF 0–12 months	+ 2.4 ± 2.2	+ 6.1 ± 3.7	**0.009**
ΔLV GLS 0–12 months	−2.6 ± 1.3	−4.7 ± 2.0	**< 0.001**
ΔPSD (ms)	−28.1 ± 15.7	−42.3 ± 19.6	0.062
ΔLVMD (ms)	−19.4 ± 12.9	−31.6 ± 17.4	0.068

Table 4 shows that an LTTAD ≥ 21.5 mm is associated with a greater improvement in LVEF and LV GLS over 12 months (*P* = 0.009 and *P* < 0.001, respectively). This indicates that a more distal lead position does not negatively impact LVEF or LV GLS improvement. This is further supported by the analysis in Table 4, which shows that patients with an LTTAD ≥ 21.5 mm had a clinically greater reduction in PSD and LVMD compared to the LTTAD < 21.5 mm group (ΔPSD − 42.3 ± 19.6 ms vs. − 28.1 ± 15.7 ms; *P* = 0.062 and ΔLVMD − 31.6 ± 17.4 ms vs. − 19.4 ± 12.9 ms; *P* = 0.068). These findings suggest a trend indicating that pacing more distally from the tricuspid annulus does not result in worse intraventricular synchrony in LBBAP implantation.

The results of the multivariate logistic regression analysis (Table 5) show that baseline QRS duration and baseline LVEF are the main predictors of improvement in left ventricular function. A wider baseline QRS duration was significantly associated with an increased likelihood of LVEF improvement (Model 1: OR 1.29; 95% CI 1.06–1.58; *p* = 0.011), indicating that patients with more severe electrical dyssynchrony prior to implantation have greater potential for improvement through physiological resynchronization with CSP implantation.

**Table 5:** Multivariate Logistic Regression Analysis Results Between QRS Duration and Baseline LVEF on Improvement of Left Ventricular Function

Variable	OR	95% CI	*p*
Model 1			
QRS baseline (ms)	1.29	1.06–1.58	0.011
LVEF baseline (%)	0.88	0.80–0.96	0.005
Model 2			
QRS baseline (ms)	1.27	1.04–1.56	0.015
LVEF baseline (%)	0.91	0.79–1.03	0.057
Age	0.997	0.96–1.03	0.792
Hypertension	1.65	0.47–5.76	0.432
Diabetes mellitus	0.52	0.17–1.57	0.248
Coronary artery disease	1.23	0.37–3.71	0.561
Heart Failure	2.62	0.76–9.00	0.127
Model 3			
QRS baseline (ms)	1.28	1.05–1.55	0.013
LVEF baseline (%)	0.81	0.79–0.83	0.006
VP burden > 20%	2.65	0.83–8.42	0.098
Dual chamber (vs. single)	1.84	0.67–5.08	0.241
Model 4			
QRS baseline (ms)	1.31	1.09–1.60	0.007
LVEF baseline (%)	0.88	0.79–0.97	0.026
LBBAP vs. HBP	1.73	0.89–3.61	0.143

Similarly, a lower baseline LVEF was correlated with a higher likelihood of improvement (OR 0.88; 95% CI 0.80–0.96; *p* = 0.005), suggesting that patients with more severe left ventricular dysfunction at baseline have more room for recovery following therapy. In models that included additional clinical (Model 2) and technical variables (Model 3), baseline QRS duration and LVEF remained consistent as significant predictors. Other variables such as age, hypertension, diabetes, coronary artery disease, VP burden, and device type did not show a significant association with improvement in left ventricular function. In Model 4, which included the pacing type variable (LBBAP vs. HBP), the association between baseline QRS duration and baseline LVEF remained significant, while LBBAP showed a positive trend toward LVEF improvement (OR 1.73; 95% CI 0.83–3.61), although it did not reach statistical significance (*P* = 0.143).

## Discussion

Based on the analysis of this study, several important findings were identified: (1) Both CSP approaches, HBP and LBBAP, were associated with improvements in LVEF and intraventricular synchrony; (2) Pacemaker parameters showed favorable profiles, with lower threshold and higher R-wave amplitude in line with LBBAP implantation, and shorter QRS duration consistent with HBP implantation; (3) A more distal lead placement in LBBAP was not inferior compared to proximal lead positioning.

In this study LBBAP there was a significant difference in LVEF improvement between the HBP and LBBAP methods. Several factors may have contributed to this finding. First, patients in the HBP group did not have indications for cardiac resynchronization therapy due to the absence of ventricular dyssynchrony causing reduced LVEF. Therefore, improving LV function through resynchronization was not the primary goal in patients undergoing HBP implantation. Second, patients in the LBBAP group had lower baseline LVEF, which may have led them to receive more comprehensive guideline-directed medical therapy (GDMT) compared to the HBP group. Although some current guidelines recommend HBP over LBBAP, several studies have shown that LBBAP yields more favorable outcomes in terms of implantation success rates, capture thresholds, sensing amplitudes, and lead-related complications [10, 14, 15]. This is also supported by the results of the multivariate logistic regression analysis in this study, which showed that baseline LVEF is a key predictor of LVEF improvement (Table 5), indicating that patients with more severe baseline left ventricular dysfunction have greater potential for recovery following CSP implantation. This is in line with previous research stating that the higher the patient’s baseline LVEF, the smaller the remaining room for improvement in LVEF [16]. Nevertheless, other studies have shown that both HBP and LBBAP can improve left ventricular function, even when compared to biventricular pacing. However, LBBAP offers the advantage of having a more stable and lower pacing threshold while achieving similar electromechanical outcomes compared to HBP [7, 17]. 

The advantage of LBBAP in terms of electrical parameters was also evident in this study, which demonstrated a significant difference in threshold and R-wave sensing. This aligns with the known benefit of LBBAP implantation, which features lower pacing thresholds and consequently lower energy consumption compared to HBP, making LBBAP a more favorable option [10, 18]. The lower R-wave sensing observed in HBP is associated with the implantation technique. In HBP, an R-wave sensing value greater than 2 mV is one of the criteria required to ensure stable and effective pacing. In contrast, R-wave sensing in LBBAP is generally comparable to that of RV lead placement, typically greater than 4 mV. Nevertheless, LBBAP has certain limitations, one of which is the potential worsening of tricuspid regurgitation (TR) [10]. Moderate to severe tricuspid valve regurgitation (TVR) has been linked to impaired right ventricular function, a higher likelihood of developing heart failure, and reduced long-term survival outcomes [16]. 

In addition to the greater improvement in LVEF observed in the LBBAP group compared to the HBP group, a significant enhancement in LV GLS was also noted, indicating improved left ventricular function. Global longitudinal strain (GLS) is a method that can serve as a prognostic marker in cardiac resynchronization therapy and offers advantages in detecting subclinical LV dysfunction at an early stage [19, 20]. The improvement in LV GLS, which paralleled the significant increase in LVEF, is consistent with previous studies showing that LV GLS is a sensitive parameter for assessing left ventricular dysfunction [21]. Moreover, LV GLS may also have prognostic value for predicting future improvements in LVEF in both conduction system pacing (CSP) and biventricular pacing [20]. 

Another parameter in this study that showed a significant difference was QRS duration, where follow-up QRS duration in the HBP group showed a more significant shortening compared to the LBBAP group. This finding slightly differs from the meta-analysis by Abdin et al., in which 9 studies showed only minimal reductions in QRS duration in both the HBP and LBBAP groups [22]. However, upon closer examination of that meta-analysis, only one study included patients with a very wide QRS duration (> 140 ms), who showed a significant reduction in QRS duration after implantation, whereas the baseline QRS duration in this study was approximately 120 ms. Although QRS duration is one of the indicators of abnormal electrical activation patterns that can lead to intraventricular mechanical dyssynchrony and reduced pump efficiency and cardiac performance,^22^ the LVEF in the LBBAP group still improved compared to baseline. This is consistent with previous studies indicating that not all wide QRS complexes result in reduced cardiac output, and in such cases, resynchronization therapy can still improve left ventricular function even in patients with a narrow baseline QRS duration [15, 23]. Moreover, although conduction abnormalities are commonly observed, this is not always the case. Patients with heart failure may experience left ventricular (LV) dyssynchrony even in the absence of regionally delayed electrical activation. Patients with systolic heart failure and a narrow QRS complex have been shown to exhibit mechanical dyssynchrony with a prevalence ranging from 30% to 50%.^22^ Nevertheless, the multivariate logistic regression analysis also demonstrated that QRS duration is a significant predictor, alongside baseline LVEF, of greater LVEF improvement. This indicates that a wider QRS duration reflects the presence of intraventricular dyssynchrony, which negatively affects cardiac performance [24]. 

An interesting finding in this study is that a longer Lead to Tricuspid Annulus Distance (LTTAD) ≥ 21.5 mm was associated with an improvement in LVEF of more than 5% in the LBBAP group. This indicates that a more distal implantation in LBBAP does not demonstrate inferiority in terms of intraventricular synchrony. As is known, one of the advantages of using LBBAP is the broader area available for implantation; however, more distal pacing may lead to rapid LV activation and delayed antegrade activation of the right bundle branch (RBB), which can affect cardiac function [9]. This is supported by the study by Chaodi et al., which compared ventricular synchrony in heart failure patients among LBBP, LFP, and LVSP, showing that there was no difference in either electrophysiological or mechanical synchrony between proximal and distal pacing of the left conduction system. However, it was also noted that LBBAP with confirmed LBB capture still demonstrated superior electrophysiological performance compared to pacing without LBB capture [8]. 

This may alleviate concerns that more distal LBBAP implantation could negatively affect interventricular synchrony, as, conversely, a more proximal implantation may increase the risk of worsening tricuspid regurgitation, which can impair right ventricular function [16]. LBBAP implantation has been associated with a potential worsening of tricuspid valve regurgitation, observed in up to one-third of patients undergoing the procedure, particularly when the lead is positioned basally. In contrast, this complication is rarely seen with HBP implantation [10]. This finding is supported by a meta-analysis by Karwiky et al., which reported that shorter LTTAD was associated with worsening tricuspid regurgitation (TR). The studies included in that meta-analysis identified cut-off values for LTTAD ranging from 16.1 to 24.5 mm, with an average of 19.9 mm. Based on these findings, a lead-to-annulus distance of at least approximately 20 mm may help reduce the risk of TR deterioration [12]. 

In addition, the analysis in this study demonstrated that left ventricular mechanical dyssynchrony (LVMD) and peak strain dispersion (PSD), both echocardiographic parameters used to assess dyssynchrony, showed improvement with both CSP methods (HBP and LBBAP) even though no significant difference was observed between the two. LVMD reflects the degree of ventricular contractile discoordination and has prognostic value for heart failure outcomes. In patients undergoing cardiac resynchronization therapy (CRT), LVMD is considered one of the parameters for assessing therapeutic success. LVMD is evaluated by measuring the standard deviation of the time to peak regional longitudinal strain (PSD). Therefore, a reduction in PSD corresponds to a decrease in LVMD, indicating an improvement in left ventricular dyssynchrony [25, 26]. This demonstrates that CSP is a promising approach to reduce the harmful effects of right ventricular pacing namely electrical and mechanical dyssynchrony and serves as a potential solution to the limitations associated with biventricular pacing (BiVP) [27, 28]. 

Additionally, this study also demonstrated that the choice between HBP and LBBAP did not significantly affect left ventricular function, as indicated by improvements in LVEF or QRS duration (Table 4). Therefore, the selection of pacing method should be individualized based on the specific indications and characteristics of each patient. However, LBBAP may be considered due to its high success rate, lower pacing thresholds, and broader applicability in patients with infranodal conduction disease or heart failure while taking into account the LTTAD to prevent worsening of tricuspid regurgitation associated with LBBAP implantation [6, 7, 12]. 

### Clinical implications

LBBAP is a promising alternative to HBP. A more distal lead placement in LBBAP is not inferior to a more proximal lead placement and even offers the advantage of reducing the risk of tricuspid regurgitation (TR) deterioration that may occur with a more proximal lead position in LBBAP. Additionally, LBBAP offers several advantages, including lower pacing thresholds, easier lead implantation, and higher implantation success rates compared to HBP. Nevertheless, personalized patient evaluation and ongoing surveillance of long-term outcomes are essential as this pacing strategy continues to develop and gain wider clinical application.

### Future directions and limitations

According to the European Heart Rhythm Association (EHRA), the term Left Bundle Branch Area Pacing (LBBAP) includes Left Bundle Branch Pacing (LBBP), Left Septal Pacing (LSP), and Left Ventricular Septal Pacing (LVSP), which are defined based on the anatomical lead location and specific QRS morphologies [13]. Future studies incorporating this more detailed classification could allow for a more nuanced analysis of pacing outcomes. In this study, lead to tricuspid annulus distance (LTTAD) was measured using two-dimensional echocardiography; however, this method is not considered the gold standard. The use of three-dimensional echocardiography combined with fluoroscopy could have yielded more precise measurements [29]. This research was conducted retrospectively at a single university hospital involving multiple operators; nonetheless, a standardized implantation protocol was consistently followed. Left ventricular global longitudinal strain (LV GLS) was used to assess subclinical LV dysfunction. However, echocardiographic evaluation of LV function is highly operator-dependent, which could introduce measurement variability. Additionally, a one-year follow-up may not be sufficient to assess the full long-term impact of CSP techniques. The study’s single-center design and relatively small sample size further limit the generalizability of the findings, highlighting the need for larger, multicenter trials with extended follow-up periods.

## Conclusion

Cardiac conduction system pacing, including both HBP and LBBAP, offers advantages in preventing electrical and mechanical dyssynchrony compared to right ventricular (RV) pacing, which has been associated with left ventricular (LV) dysfunction. However, LBBAP with a more distal lead placement, namely 21.5 mm from the tricuspid annulus, has shown no inferiority compared to more proximal implantation, indicating that a more distal lead position does not result in delayed activation that affects interventricular synchrony. Lead placement in LBBAP not only contributes to the prevention of electrical and mechanical dyssynchrony but also offers benefits in reducing the progression of tricuspid regurgitation (TR), which can impair right ventricular function. Considering optimal lead positioning in LBBAP may serve as a guide in selecting conduction system pacing (CSP) strategies, offering higher procedural success rates, easier lead implantation, and lower pacing thresholds compared to HBP.

## Data Availability

Data is available upon reasonable request to the corresponding author.
